# Next-generation sequencing analysis of endometrial screening liquid-based cytology specimens: a comparative study to tissue specimens

**DOI:** 10.1186/s12920-020-00753-6

**Published:** 2020-07-11

**Authors:** Toshiaki Akahane, Ikumi Kitazono, Shintaro Yanazume, Masaki Kamio, Shinichi Togami, Ippei Sakamoto, Sachio Nohara, Seiya Yokoyama, Hiroaki Kobayashi, Tsubasa Hiraki, Shinsuke Suzuki, Shinichi Ueno, Akihide Tanimoto

**Affiliations:** 1 Department of Pathology, Kagoshima University Graduate School of Medical and Dental Sciences, 8-35-1 Sakuragaoka, Kagoshima, 890-8544 Japan; 2 Center for Human Genome and Gene Analysis, Kagoshima University Hospital, 8-35-1 Sakuragaoka, Kagoshima, 890-8544 Japan; 3 Department of Obstetrics and Gynecology, Kagoshima University Graduate School of Medical and Dental Sciences, 8-35-1 Sakuragaoka, Kagoshima, 890-8544 Japan; 4 Kagoshima University Hospital Cancer Center, Kagoshima University Hospital, 8-35-1 Sakuragaoka, Kagoshima, 890-8544 Japan; 5 Department of Biomedical Informatics, Mitsubishi Space Software, 5-4-36 Tukaguchi Honmachi, Amagasaki, Hyogo 661-0001 Japan

**Keywords:** Endometrial cytology, LBC, NGS, Endometrial cancer, MSI, TMB

## Abstract

**Background:**

Liquid-based cytology (LBC) is now a widely used method for cytologic screening and cancer diagnosis. Since the cells are fixed with alcohol-based fixatives, and the specimens are stored in a liquid condition, LBC specimens are suitable for genetic analyses.

**Methods:**

Here, we established a small cancer gene panel, including 60 genes and 17 microsatellite markers for next-generation sequencing, and applied to residual LBC specimens obtained by endometrial cancer screening to compare with corresponding formalin-fixed paraffin-embedded (FFPE) tissues.

**Results:**

A total of 49 FFPE and LBC specimens (n = 24) were analyzed, revealing characteristic mutations for endometrial cancer, including *PTEN, CTNNB1, PIK3CA,* and *PIK3R1* mutations. Eight cases had higher scores for both tumor mutation burden (TMB) and microsatellite instability (MSI), which agree with defective mismatch repair (MMR) protein expression. Paired endometrial LBC, and biopsied and/or resected FFPE tissues from 7 cases, presented almost identical mutations, TMB, and MSI profiles in all cases.

**Conclusion:**

These findings demonstrate that our ad hoc cancer gene panel enabled the detection of therapeutically actionable gene mutations in endometrial LBC and FFPE specimens. Endometrial cancer LBC specimens offer an alternative and affordable source of molecular testing materials.

## Background

Endometrial cancer is the most common gynecologic cancer worldwide, affecting over 500,000 women every year [1]. Since lifestyle changes can affect the risk of developing endometrial cancer, an epidemiological approach is essential to manage the disease [2]. Moreover, genome-wide association studies have identified new susceptibility loci for endometrial cancer in the human genome, providing candidate genes for further studies aiming to unravel the mechanisms driving carcinogenesis, which could offer new opportunities for early screening and identification of therapeutic targets [3]. Unlike squamous cell carcinoma of the uterine cervix, which is characterized by pathogenesis closely related to that of human papilloma virus infection [4], endometrial carcinogenesis is linked to common cancer gene mutations and genome instability [5, 6].

Cytology specimens, including conventional smear, cytospin, liquid-based cytology (LBC), and cell block, are valuable sources of routine molecular diagnostics [7]. Extensive studies have been reported that a variety of cytology materials, containing LBC specimens suitable for next-generation sequencing (NSG) testing, especially in non-small cell lung cancer [7–12]. Meanwhile, the endometrial cytology is not only a safe and easy clinical procedure but also comparable to suction endometrial biopsy for detecting atypical endometrial hyperplasia and cancers [13–16]. Since 1987, in Japan, a cytologic examination for endometrial cancer screening has been established by the National Health Insurance law. After that, endometrial cytology, using a specific device, was introduced as a less invasive and less painful tool [17, 18] to screen high-risk population and perimenopausal women with abnormal genital bleeding. Conventional endometrial cytology preparation, however, has some disadvantages such as bloody background, cellular overlapping, and thick cell clusters; therefore, the resulting cytological screening would not be widely accepted in endometrial cancer management except in Japan [19]. After the introduction of LBC in endometrial cytology in combination with transvaginal sonography, LBC-based endometrial cytology, a less invasive and expensive procedure, was proposed to screen cancer also in asymptomatic postmenopausal women [20, 21].

To date, LBC represents a routine endometrial cytology method for cancer diagnosis and gene analysis [22]. We also previously reported that residual LBC samples could better preserve the genome quality for downstream NSG, as compared to formalin-fixed paraffin-embedded (FFPE) tissues, even after several years of storage [23]. In the present study, we established a small custom cancer gene panel with full exon coverage of 60 cancer-related genes and 17 microsatellite foci. We then used this custom panel to comparatively analyze the genetic profiles obtained from residual endometrial LBC specimens and corresponding FFPE tissues with respect to the presence of somatic gene mutations, tumor mutation burden (TMB), and microsatellite instability (MSI). We further conducted immunohistochemistry for detecting the expression of mismatch repair (MMR) proteins in the FFPE sections to compare the results obtained by genomic analysis. The detection of MMR protein expression is known as a reliable proxy of the MSI status [24]. This study demonstrates the feasibility of using a small NGS-based cancer gene panel to screen for endometrial cancer in residual LBC specimens. The reliability of the results was compared with those obtained from biopsied and resected FFPE tissue analysis.

## Methods

### Histological, cytological and blood specimens

In this study, 53 tissue and cytology samples from 24 patients with endometrial cancer registered in the Clinical Research of Cancer Gene Panel Analysis of Gynecologic Cancers study from January to August 2019 at Kagoshima University Hospital were used. The specimens included 2 mL of whole blood as a reference, FFPE tissues from 10 biopsied, 29 resected tissues (39 FFPE tissues in total), and 10 residual LBC samples of endometrial cytology. The tissues were fixed with phosphate-buffered neutral 10% formalin within 24 h and routinely processed for paraffin embedding, followed by sectioning for hematoxylin and eosin (H&E) staining. The FFPE blocks were stored in the dark at room temperature (25 ± 2 °C). The endometrial cytology samples were obtained using a specific device (Endocyte® sampler, Laboratoire CCD, Paris, France) [17, 18], and collected cells were fixed with CytoRich Red solution (Becton Dickinson, Franklin Lakes, NJ, USA). The cytology samples were routinely processed for LBC using BD SurePath liquid-based Pap Test System (Becton Dickinson). The residual LBC specimens were stored at 4 °C.

The pathological diagnosis was made by two board-certified pathologists (IK and AT), according to the World Health Organization classification [25], and the tumor fraction in the sections (% area) was evaluated in 10% increments. Similarly, the cytological diagnosis was made by two board-certified cytopathologists (IK and TH), according to the classification system proposed by the Japanese Society of Clinical Cytology [26]. For cytology specimens, atypical or tumor cell numbers were counted in five high-power fields (HPF) at × 400 magnification. The average counts were calculated and the total tumor cells (T) subjected to NGS were estimated. Similarly, non-tumor cell counts were calculated as the average of five HPF, and total non-tumor cells (N) were estimated, and then the tumor cell ratio (T/N + T) was calculated. The cytological and pathological studies were performed independently from the molecular studies described below.

### DNA extraction and quality check

Whole blood DNA was extracted using the QIAamp DNA Blood Mini Kit (Qiagen). DNA from FFPE tissues was obtained from 3 to 6 sections with 10-μm thickness, including more than 30% of the cancer area, or after microdissection using a microdissection laser (Leica Biosystems, Nussloch GmbH, Germany). One mL of residual LBC samples was centrifuged (× 12,000 *g*), and the pellets were resuspended in 95% ethanol and air-dried for 5 min. Both the FFPE sections and LBC pellets were then incubated with proteinase K (Promega, Madison, WI, USA) for 15 h at 70 °C, followed by a 1-h incubation at 98 °C in lysis buffer (Promega). After centrifugation (× 12,000 *g*), the supernatants were applied to the Maxwell RSC DNA FFPE kit and Maxwell 16 system (Promega). After measuring the extracted DNA concentration using the Qubit 3.0 dsDNA BR assay kit (Life Technologies, Grand Island, NY, USA), DNA qualities were monitored using the QIAseq DNA quantimize kit (Qiagen). A quality check score (QC score) less than 0.04 was considered as a high-quality DNA, according to our previous report [23].

### Design of the custom gene panel

A total of 60 cancer-related genes and 17 microsatellite foci [27] were selected from QIAseq Targeted DNA Custom Panel (Qiagen [28]), including 2615 primers for the regions of interest, with an average exon coverage of 99.87%. The cancer-related genes were used for the construction of a custom panel (detailed in Table 1). The selection of these 60 genes was validated according to the Catalogue Of Somatic Mutations In Cancer (COSMIC ver. 90, https://cancer.sanger.ac.uk/cosmic) to include target genes of molecular targeting drugs for solid cancers, and genes frequently expressed in ovarian and endometrial cancers.

**Table 1:** Region of interests of custom panel

60 genes				17 MSI regions		
				Target	Loci	Gene
AKT1	CTNNB1	MAP2K1	PIK3CA	Bat-25	chr4:55598208–55,598,241	KIT
APC	DDR2	MAP2K2	PIK3R1	Bat-26	chr2:47641403–47,641,591	MSH2
ATM	EGFR	MAP2K4	PMS2	MONO-27	chr2:39564890–39,564,926	MAP4K3
AR	ERBB2	MED12	PTEN	NR-21	chr14:23652343–23,652,372	SLC7A8
ARID1A	ERBB3	MET	RB1	NR-24	chr2:95849358–95,849,389	ZNF2
BARD1	ERBB4	MDM2	RAD51	MSI-1	chr1:201754407–201,754,432	NAV1
BRAF	ESR1	MLH1	STK11	MSI-3	chr2:62063090–62,063,115	FAN161A
BRCA1	FGFR1	MSH2	TP53	MSI-4	chr2:108479619–108,479,675	RGPD4
BRCA2	FGFR2	MSH6	RET	MSI-6	chr5:172421757–172,421,780	ATP6V0E1
BRIP1	FGFR3	MTOR		MSI-7	chr6:142691947–142,691,972	GPR126
CCND1	FLT3	MUTYH		MSI-8	chr7:1787516–1,787,541	ELFN1
CD274	HRAS	MYC		MSI-11	chr11:106695511–106,695,531	GUCY1A2
CDK4	IDH1	NF1		HSPH1-T17	chr13:31722617–31,722,642	HSPH1
CDK6	IDH2	NRAS		MSI-12	chr15:45897768–45,897,790	BLOC1S6
CDKN2A	KDR	PALB2		MSI-13	chr16:18882656–18,882,679	SMG1
CDH1	KIT	PDCD1		MSI-14	chr17:19314914–19,314,940	RNF112
CTLA4	KRAS	PDGFRA		EWSR1	chr22:29696465–29,696,489	EWSR1

### NGS analysis

Forty ng of DNA from whole blood cells and LBC specimens and 100–200 ng of DNA from FFPE tissue sections were used to construct the NGS library based on QIAseq Targeted DNA Custom Panel (Qiagen). The quality of the libraries was monitored using an Agilent high-sensitivity DNA kit (Agilent Technologies, Santa Clara, CA, USA) to confirm the successful generation of 300-bp PCR products. The libraries were then applied to MiSeq sequencer (Illumina, San Diego, CA, USA) after dilution with a hybridization buffer to a final concentration of 20 pM. The obtained sequencing data were analyzed by the Qiagen Web Portal service (https://www.qiagen.com/us/shop/genes-and-pathways/data-analysis-center-overview-page/ ). Qiagen Web Portal service, equipped with a smCounter analyzing pipeline [29], indicates that a mean unique molecular index (UMI) sequence depth of 2,000 is necessary for detection of 0.5% variant allele frequency (VAF), and the cut-off value of VAF varies, inversely proportional to the mean sequence depth. Our NGS, with a condition of tumor fraction ≥20% and cluster passing filter rate ≥ 90%, yielding a mean UMI depth of 500, at which the SNP detection noise was low when VAF was ≥10%. Therefore, the cut-off value of VAF was set to 10%, also considering the sample loading size in one NGS run and its cost. Human genome reference GRCh37 hg19 (https://www.ncbi.nlm.nih.gov/assembly/GCF_000001405.13/) and COSMIC database were used as the analytical references. For validation of the NSG panel, all the sequence data were analyzed and annotated by Qiagen Web Portal service and Mitsubishi Space Software (Amagasaki, Hyogo, Japan, https://www.mss.co.jp/business/life-science/). Human cell lines (HEK293T [catalogue # ATCC CRL-3216], HCT166 [catalogue # ATCC CCL-247], and MDA-MB453 cells [catalogue # ATCC HTB-131]) obtained from American Type Culture Collection (Rockville, MD) were analyzed as controls, and the genomic sequence data were compared to those deposited in the COSMIC database. In this study, DNA sequence data obtained from whole blood were used only for reference, whereas germline analysis was not performed.

### Calculation of TMB and MSI scores

The numbers of missense mutations, including non-synonymous mutations and internal deletions, with more than 10% VAF, were counted as somatic mutations. TMB was calculated as the number of single nucleotide variants/Mbp of DNA sequence [30, 31]. MSI scores were determined by MSIsensor (ver. 1.0) [32, 33].

### Immunohistochemistry for MMR proteins

To evaluate the expression of MMR proteins, FFPE tissue sections were applied to immunohistochemistry (IHC) using antibodies against MLH1 (clone ES05 M3640), MSH2 (clone FE11 M3639), MSH6 (clone EP49 M3646) and PMS2 (clone EP51 M3647) purchased from DAKO (Tokyo, Japan) [34, 35]. Staining was performed on representative 5-μm-thick FFPE sections with Envision FLEX High pH K8000 system (DAKO) according to the manufacturer’s protocol. Positive nuclear staining of lymphocytes in the tissue sections was monitored as a positive control. MMR deficiency was defined as complete loss of nuclear staining for both MLH1 and PMS2, both MSH2 and MSH6, MSH6 only, or PMS2 only. When partial loss of each protein was observed, the case was defined as showing heterogeneous expression.

### Statistical analyses

All values are expressed as the mean ± standard deviation. Significant differences were analyzed using Welch’s *t*-test and Mann-Whitney U test. Values of *p* < 0.05 were considered statistically significant. The cut-off values for the evaluation of TMB-high and MSI-high conditions were determined by receiver operator characteristic (ROC) curves.

### Ethical approval for the genome studies

The studies using clinical samples were approved by the Ethics Committees for Clinical and Epidemiologic Research at Kagoshima University, and written-informed consent was obtained from each participant. The studies did not include participants younger than the age of 20.

## Results

### DNA quality and quantity obtained from FFPE and LBC specimens

The results of the pathological diagnosis, DNA quality, and input DNA for NGS of the 24 endometrial cancer cases are summarized in Table 2. The storage period of FFPE and LBC specimens ranged from 2 weeks to 3 years. The tumor fraction of the FFPE sections ranged from 30 to 90%. In endometrial LBC specimens, estimated tumor cell count (T) subjected to NGS varied from 1500 to 140,000, and the tumor cell ratio varied from 27 to 95% (T/N + T). All of the genomic DNA extracted from the FFPE and LBC samples demonstrated high-quality and sufficient quantity for library construction and successful sequencing.

**Table 2:** Pathological and DNA sample information for NGS analysis

Case no.	Sample no.	Samples	Pathological diagnosis	Storage time	DNA yield (ng/μL)	QC score	Input DNA (ng)	Tumor fraction in FFPE (%)	Tumor cell count and tumor cell ratio in LBC			Insufficient VAF call
									Tumor cells (T)	Non-tumor cells (N)	Tumor cell ratio (T/N+T)%	
**1**	**1**	SR(Ut)	EC G1	1 m	1.655	0.012	200	30				
**2**	**2**	SR(Ut)	EC G1	2 m	5.510	0.003	100	80				
**3**	**3**	SR(Ut)	EC G2	1 m	2.980	0.008	100	60				
**4**	**4**	SR(Ut)	EC G1	1 m	1.521	0.016	200	50				
**5**	**5**	SR(Ut)	EC G1	3 m	4.033	0.010	119	80				
**6**	**6**	SR(Ut)	EC G1	3 m	6.636	0.003	72	30				
	**7**	SR(Ut)	EC G2	3 m	6.071	0.004	79	60				
**7**	**8**	SR(Ut)	EC G1 + Sq	1 m	5.912	0.003	200	70				
**8**	**9**	SR(Ut)	Mixed EC/SC	2 m	3.487	0.010	138	50				
	**10**	SR(Om)	Mixed EC/SC	2 m	4.897	0.007	98	30				
**9**	**11**	Bx	SC	3 m	6.425	0.004	75	80				
	**12**	SR(Ut)	CCC	3 m	1.930	0.015	168	90				
	**13**	End LBC	AC	3 m	28.879	0.000	20		6661	10,059	39.8	
**10**	**14**	SR(Ut)	DC	3 m	3.337	0.007	144	70				
	**15**	SR(Ut)	EC G1	3 m	5.743	0.004	84	70				
**11**	**16**	SR(Ut)	AC	1 m	2.327	0.011	168	60				
	**17**	SR(Ut)	DC	1 m	0.939	0.016	168	50				
	**18**	SR(Ov)	EC G3	1 m	1.778	0.012	168	40				
**12**	**19**	Bx	AC	1 m	2.264	0.010	168	60				
	**20**	SR(Ut)	EC G2	1 m	3.025	0.006	168	70				
	**21**	End LBC	Malig	2 m	21.861	0.002	22		19,588	4087	82.7	
**13**	**22**	SR(Ut)	EC G1	1 m	2.338	0.010	168	50				
**14**	**23**	Bx	EC G1	1 m	5.664	0.001	85	70				
	**24**	End LBC	AGC	2 m	23.839	−0.003	10		1450	136	91.4	
**15**	**25**	SR(Ut)	DC	1 m	6.249	0.008	77	90				
	**26**	SR(Ut)	AC	1 m	5.830	0.010	85	90				
**16**	**27**	Bx	AC	1 m	5.029	0.010	95	80				
	**28**	SR(Ut)	Serous	1 m	0.872	0.017	168	40				
	**29**	End LBC	AC	2 m	14.257	0.005	31		22,261	3928	85.0	
**17**	**30**	SR(Ut)	AH	1 m	3.443	0.007	140	70				
	**31**	SR(Ut)	EC G1	1 m	4.137	0.006	116	60				
**18**	**32**	Bx	EC G1	1 m	3.794	0.009	127	90				
	**33**	SR(Ut)	EC G1	1 m	3.025	0.012	168	80				
	**34**	End LBC	EC	2 m	3.392	0.008	11		8043	20,911	27.8	*
**19**	**35**	SR(Ut)	EC G1 + Sq	2 m	4.585	0.004	105	30				
	**36**	End LBC	EC	4 m	16.984	0.000	26		7739	9261	45.5	
**20**	**37**	Bx	EC G2	3y	0.492	0.022	168	60				
**21**	**38**	Bx	EC G1	3 m	2.612	0.012	168	90				
	**39**	SR(Ut)	EC G1	1 m	1.321	0.025	154	90				
	**40**	End LBC	EC	3 m	16.114	0.005	36		16,855	10,739	61.1	
**22**	**41**	Bx	EC G3	2y	1.024	0.022	168	40				
	**42**	End LBC	AGC	1 m	16.208	0.004	30		14,816	1019	93.6	
**23**	**43**	Bx	AC	2 m	3.479	0.010	138	90				
	**44**	SR(Ut)	EC G2	2w	2.443	0.012	168	90				
	**45**	End LBC	EC	2 m	8.576	0.004	54		45,220	2537	94.7	
**24**	**46**	Bx	EC	4 m	2.443	0.009	168	80				
	**47**	SR(Ut)	EC G1	2 m	5.058	0.005	95	80				
	**48**	SR(Ov)	EC G1	2 m	1.694	0.011	168	70				
	**49**	End LBC	AC	4 m	24.088	−0.001	31		21,930	6661	76.7	

*SR*surgical resection,*Bx*Endometrial biopsy,*LBC*liquid-based cytology,*End*Endometrial,*Ut*Uterus,*Om*Omentum*Ov*Ovary,*EC*Endometrioid carcinoma,*SC*Serous carcinoma,*CCC*Clear cell carcinoma,*AC*Adenocarcinoma,*Sq*Squamous differentiation,*DC*Dedifferentiated carcinoma,*Malig*Malignant cell,*AGC*Atypical glandular cell,*AH*Atypical hyperplasia,*w*, week,*m*month*y*, year

### Mutations detected in FFPE specimens

The detected mutations in endometrial cancers and genomic information of the variants are summarized in supplemental Tables S1 and S2. Among the 24 cases, 18 were finally diagnosed as endometrioid carcinoma (EC) by biopsy or surgical resection, including 12 cases of G1, 5 cases of G2, and 1 case of G3 EC. The other 6 cases consisted of 3 dedifferentiated carcinomas (DC), and 1 case each of mixed EC/serous carcinoma (SC), SC, and clear cell carcinoma (CCC). These endometrial cancers showed common mutation profiles, including *PTEN, CTNNB1, PIK3CA,* and *PIK3R1* mutations.

The cases of mixed EC/SC, CCC, and SC had additional *TP53* mutations. Two different FFPE sections relative to case no. 6 were analyzed, revealing G1 and G2 EC. While NGS analysis unveiled common mutations in *PTEN*, *CTNNB1*, and *ARID1A*, different *PIK3CA* and *PIK3R1* mutations were also detected, suggesting the existence of at least two cancer clones. The three DC cases harbored *PTEN*, *CTNNB1*, *PIK3CA*, or *PIK3R1* mutations along with multiple mutations in receptor-type tyrosine kinase genes, such as *FGFR, ERBB, RET,* and *FLT*. The mutation profiles of the EC and DC sections were not completely comparable but exhibited similarity with respect to *PTEN* mutation.

### Mutations detected in LBC specimens

Endometrial LBC specimens contained abundant atypical cells, resulting in a higher frequency of mutation detection in the endometrial LBC specimens (9 out of 10 cases; supplemental Table S1). Mutations in *PTEN*, *CTNNB1*, and *PIK3CA* were identified in 1 case of atypical cell cytology (case no. 14), in which the diagnosis of G1 EC was confirmed by endometrial curettage biopsy.

### Relations between MMR protein expression, TMB, and MSI

**Fig. 1:**
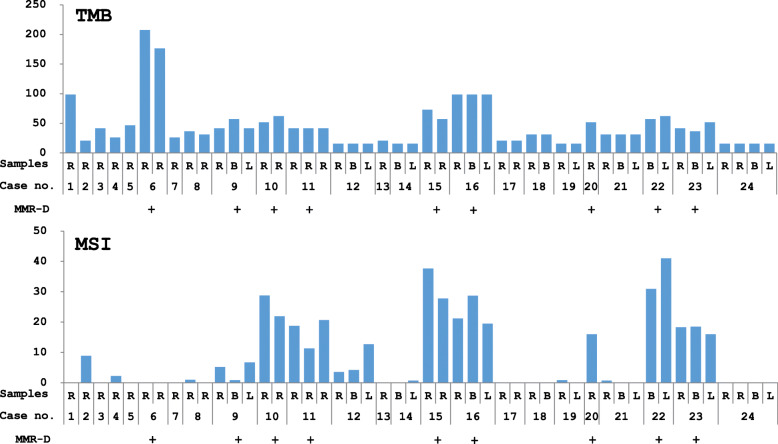
TMB and MSI scores in each case of endometrial cancer. Scores for TMB and MSI were calculated from the NGS analysis of FFPE tissues obtained by surgical resection (R), biopsy (B), and by LBC specimens (L). Most of the MMR protein-deficient cases (MMR-D: +), screened by IHC, had both higher TMB and MSI scores. In case nos. 1, 3, 5, and 12, no MMR-D was detected despite higher TMB or MSI

**Fig. 2:**
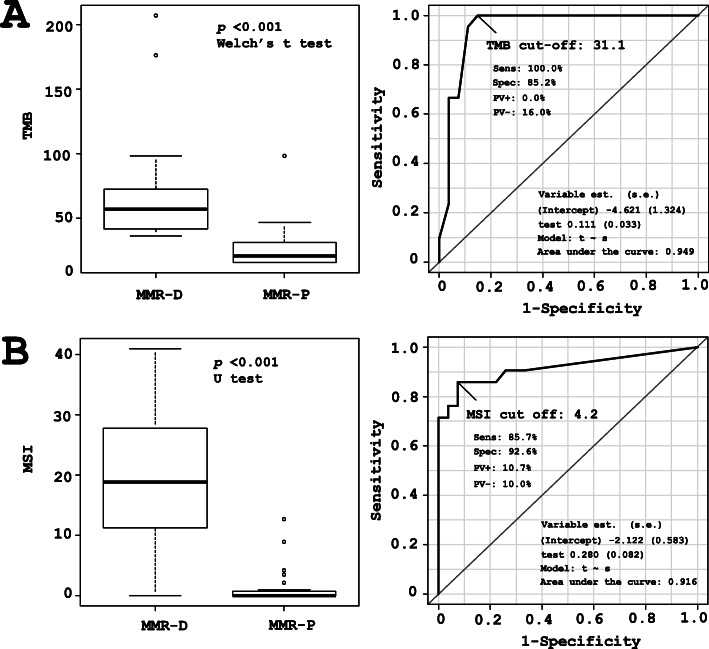
Relation of TMB, MSI scores, and MMR protein expression. **a** TMB scores and ROC curve. **b, c, d** MSI score and ROC curve. Cases with positive MMR protein expression (MMR-P) exhibited lower TMB and MSI scores, but the MMR protein-deficient cases (MMR-D) significantly demonstrated higher scores for TMB (*p* < 0.001) and MSI (*p* < 0.001). The ROC curve was used to determine the cut-off scores for TMB-H and MSI-H as 31.1 and 4.2, respectively

The overall relationships between MMR protein deficiency (MMR-D), TMB, and MSI status are shown (Fig. 1). In cases of MMR-D (9 cases, 21 samples), the TMB score was significantly higher than in cases of MMR protein proficiency (MMR-P) (15 cases, 27 samples) (*p* < 0.001; Fig. 2a, left). The MSI score of MMR-D cases was also significantly higher than that in MMR-P cases (*p* < 0.001; Fig. 2b, left). Estimated cut-off values for the evaluation of TMB-high (TMB-H) and MSI-high (MSI-H) were > 31.1 and > 4.2, respectively, as determined by the ROC curve (Fig. 2a and b).

**Fig. 3:**
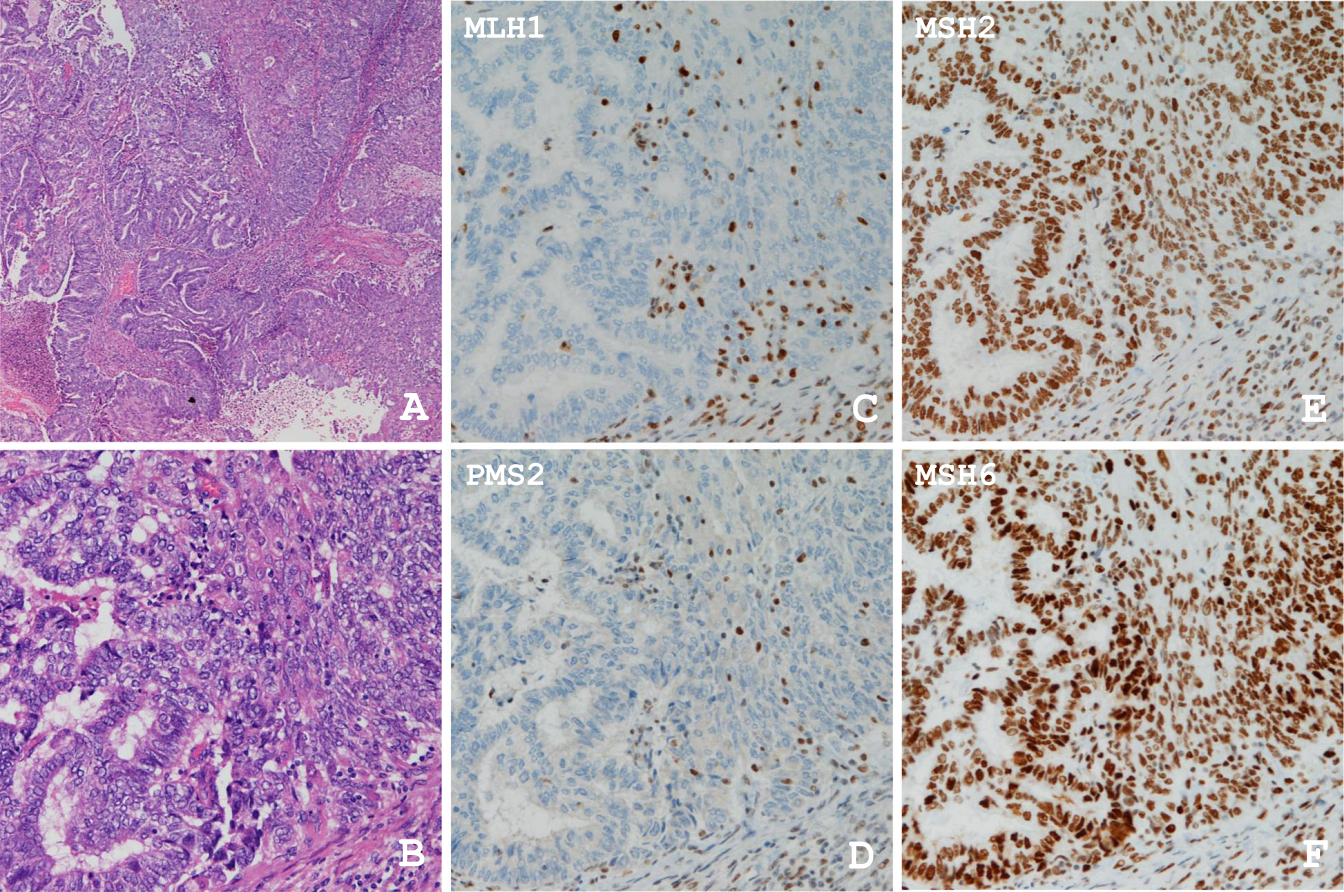
Representative H&E sections and IHC for MMR protein expression. **a** Scanning view of endometrioid carcinoma G2 (H&E, original magnification: 40×). **b** Higher power view of the endometrioid carcinoma G2 arranged in solid and glandular patterns (H&E, original magnification: 200×). **c** Absent expression of MLH1 in both the glandular and solid components (IHC, original magnification 200×). **d** Absent expression of PMS2 (IHC, original magnification 200×). **e** MSH2 expression was noted in both components (IHC, original magnification 200×). **f** MSH6 expression was also observed in the glandular and solid parts (IHC, original magnification 200×). Note the expression of these four proteins in stromal lymphocytes as an internal control

The IHC results, TMB, and MSI status are summarized in supplemental Table S3. The majority of MMR-D cases (8 of 9 cases), but not MMR-P cases, showed both TMB-H and MSI-H. The exception was the case no. 6, exhibiting heterogeneous loss of expression in MLH1, PMS2, and MSH6, which was only classified as TMB-H. One case with high TMB and MSI scores (case no. 16) presented a pathogenic mutation in *MSH6* and the corresponding loss of MSH6 expression. Three cases of DC with MMR-D were found to be both TMB-H and MSI-H, among which 2 cases harbored pathogenic *MLH1* mutations and loss of MLH1 and PMS2 protein expression. In 3 cases (case nos. 1, 3 and 5), no loss of MMR protein was detected despite a TMB-H status. In 1 case (case no. 12), despite being MSI-H positive, no MMR protein loss was detected. The photomicrographs of H&E staining and IHC from a representative MMR-D case of G2 EC (case no. 23) with loss of MLH1 and PMS2 expression are shown in Fig. 3. Very similar findings were obtained from cases no. 20 and 22, and both the TMB and MSI scores were over the cut-off values in these cases.

### Correlation of the genetic diagnosis from LBC and FFPE specimens

In 10 cases (case nos. 9, 12, 14, 16, 18, 19, 21–24), the FFPE tissues from biopsy and/or resection along with endometrial LBC, were subjected to gene panel analysis. For cases no. 14 and 22, biopsied and endometrial LBC samples were analyzed together, revealing the same mutations. In 8 cases (case nos. 9, 12, 16, 18, 21, 23, and 24), 3 paired endometrial LBC, biopsied, and resection FFPE specimens were available for genetic studies. Seven of these 8 cases (except for case no. 18) exhibited almost identical mutation profiles and a similar TMB/MSI status.

**Fig. 4:**
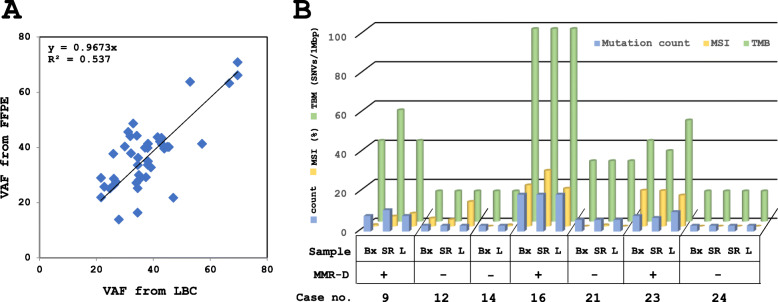
Correlation between corresponding LBC and FFPE specimens in VAF, Mutation counts, TMB, and MSI. **a** The VAF of mutated genes obtained from 7 endometrial LBC and that of the same gene mutations from corresponding FFPE were plotted, showing a good correlation between the selected LBC and FFPE specimens. The data consisted of VAF of 42 genes detected by NGS analysis in 7 cases with paired LBC and biopsy specimens. **b** Concordance of mutation counts, TMB, and MSI scores in paired LBC and FFPE (biopsy and resection) specimens from the 7 cases. Bx, biopsy; SR, surgical resection; L, LBC; MMR-D, mismatch repair protein deficiency

The VAF detected from 7 endometrial LBC specimens (case nos. 9, 12, 14, 16, 21, 23, and 24) showed a remarkably similar VAF to that of the same gene mutations from the corresponding biopsied FFPE (Fig. 4a). In case no. 22, VAF of *APC* markedly changed from 85.6% in LBC to 58.9% in biopsied FFPE, possibly because chemotherapy resulted in tumor clonal selection after LBC sampling and before the biopsy, and, therefore, the data were excluded from the Fig. 4a. All together with the results obtained from these 7 cases, a fine concordance was observed between endometrial LBC, and biopsied and/or resected FFPE tissue specimens in numbers of mutations, TMB and MSI scores, and MMR protein expression (Fig. 4b).

## Discussion

We showed that LBC specimens offered sufficient DNA quality and quantity for NGS analyses. Endometrial LBC samples contained a much higher tumor cell ratio, allowing for NGS analysis to be efficiently performed. The endometrial LBC specimen containing at least 1450 tumor cells, and 10 ng of input DNA were successfully analyzed with the present protocol. A previous report suggested that 5000 viable cells are necessary for successful NGS using a 50-cancer gene hotspot panel from conventional cytology samples [36]. Therefore, appropriate cytology specimens containing more than several thousand cells with a higher tumor ratio could be an alternative source for genetic analysis to FFPE and conventional cytology specimens. However, the use of LBC specimens for NGS analysis faces a few disadvantages [37, 38]. Unlike NGS in scraped cells from smears, in which the same cells observed by cytopathologists are subjected to molecular testing, NGS analysis from LBC specimens is started with pooled residual samples. Therefore, although we checked for the presence of tumor cells in the residual LBC specimens and then applied to DNA extraction, false-negative materials may be applied to NGS. A rapid on-site evaluation to ensure the sufficient preservation of tumor cells are unavailable in LBC specimens, either. To the best of our knowledge, another disadvantage is that an efficient method to enrich tumor cell fraction in LBC specimens has not yet been developed. In this regard, the LBC specimens, such as inflammatory cell-rich ascites, may not be always suitable for NGS. Conventional smears and cell blocks are superior to LBC for increasing tumor cell fraction by trimming techniques [38, 39]. Conversely, one of the advantages of LBC specimens is the well-preserved DNA quality and yield even after long-term storage [23]. The absence of diagnostic material loss due to cell scraping and less complicated procedures represent additional advantage of LBC specimens.

Analysis with our custom-built panel identified mutations in *PTEN* (79%), *CTNNB1* (54%), *PIK3R1* (42%), *PIK3CA* (58%), *ARID1A* (67%), and *TP53* (21%) in the FFPE specimens, and these variant frequencies agree with previous reports [40–42]. In 9 of 10 endometrial LBC samples, genetic analysis was successfully completed with *PTEN* (56%), *CTNNB1* (44%), *PIK3R1* (33%), *PIK3CA* (56%), *ARID1A* (56%), and *TP53* (33%) mutation frequencies comparable to those previously reported based on amplicon sequencing for 5 genes (*PETN, PIK3CA, CTNNB1, KRAS,* and *TP53*), in which at least one pathogenic variant was identified in 17 of 20 cases (85%) [43]. Of note, a few differences in genomic profiles among different specimens from the same patients were observed. One possible explanation would be the presence of cancer heterogeneity harboring distinct gene mutations [44]. Furthermore, in endometrial neoplastic lesions, a complex non-linear molecular evolution happens between atypical endometrial hyperplasia (AH)/endometrial intraepithelial neoplasia (EIN) and EC [45]. The presence of minor AH/EIN components might result in genomic heterogeneity. In the present study, we did not analyze AH/EIN cases. Since a significant overlap of gene mutations is reported between AH, EIN, and EC [45, 46], a combination study with cytology and pathology would further improve the diagnostic accuracy.

Since the loss of MMR protein expression can occur by methylation and mutations in the promoter region, in addition to loss-of-function mutations in the coding region, a validation of the exon sequence would not be sufficient to evaluate the MMR expression and function [47–49]. Actually, in the present study, only 3 of 9 MMR-D cases presented pathogenic mutations in *MLH1*, *MSH6*, or *PMS2*. Although IHC is considered a practical tool for speculation of TMB and MSI conditions as a first screening step [50, 51], the results of MMR protein IHC were not always comparable to those of MMR gene mutation identification. Thus, evaluation of TMB and MSI using NGS-based genome analysis along with IHC would be a practical strategy for clinical testing [52–54]. NGS analysis with endometrial LBC specimens, which were not usually used for immunocytochemistry of MMR proteins, consistently detected TMB-H and MSI-H. Therefore, our custom-made panel would possibly be beneficial to determine TMB-H and MSI-H status. However, the sample size was relatively small, and the NGS panel is also insufficient to comprehensively detect TMB and MSI at the whole-exome sequencing level, in which TMB calculation algorithm often weights down mutation data from hotspot to prevent TMB over-estimation [32, 33, 55, 56].

## Conclusion

The NGS-based panel with coverage of 60 cancer genes and 17 microsatellite foci demonstrated highly recurrent somatic mutations, TMB, and MSI in FFPE and endometrial LBC specimens. In addition, the endometrial cytology can efficiently collect cells from the entire part of the endometrial cavity to predict histological subtypes [16] and also is available to detect asymptomatic endometrial cancer by the less invasive cytologic screening [14]. Therefore, the small size cancer gene panel and endometrial LBC specimens would be an alternative tool for genetic testing as a diagnostic or therapeutic strategy. As in the case of NGS analysis from FFPE tissues, it is vital that cytopathologists correctly estimate the amounts of tumor cells, to select suitable LBC specimens for molecular diagnosis appropriately [7].

## Supplementary information

**Additional file 1: Table S1.** Somatic mutation prolife detected in endometrial cancers.

**Additional file 2: Table S2.** Genomic information of variants detected in endometrial cancers.

**Additional file 3: Table S3.** TMB, MSI and MMR protein deficiency.

## Data Availability

The datasets generated and analyzed during the current study are made available as supplementary Tables (Supplementary Tables S1, S2, and S3) that lists all qualified variants. Database used in this study were Catalogue Of Somatic Mutations In Cancer (COSMIC ver. 90, https://cancer.sanger.ac.uk/cosmic), Qiagen Web Portal service (https://www.qiagen.com/us/shop/genes-and-pathways/data-analysis-center-overview-page/), and Human genome reference GRCh37 hg19 (https://www.ncbi.nlm.nih.gov/assembly/GCF_000001405.13/).

## Abbreviations

LBC: Liquid-based cytology
FFPE: Formalin-fixed paraffin-embedded
TMB: Tumor mutation burden
MSI: Microsatellite instability
MMR: Mismatch repair
NGS: Next-generation sequencing
H&E: Hematoxylin and eosin
HPF: High-power fields
VAF: Variant allele frequency
IHC: Immunohistochemistry
ROC: Receiver operator characteristic
EC: Endometrioid carcinoma
DC: Dedifferentiated carcinoma
SC: Serous carcinoma
CCC: Clear cell carcinoma
